# Brain volumes as predictors of tDCS effects in primary progressive aphasia

**DOI:** 10.1016/j.bandl.2019.104707

**Published:** 2019-11-05

**Authors:** Vânia de Aguiar, Yi Zhao, Andreia Faria, Bronte Ficek, Kimberly T. Webster, Haley Wendt, Zeyi Wang, Argye E. Hillis, Chiadi U. Onyike, Constantine Frangakis, Brian Caffo, Kyrana Tsapkini

**Affiliations:** a Department of Neurology, Johns Hopkins School of Medicine, Baltimore, MD, United States; b Center for Language and Cognition Groningen (CLCG), University of Groningen, Netherlands; c Department of Biostatistics, Johns Hopkins School of Public Health, Baltimore, MD, United States; d Department of Radiology, Johns Hopkins School of Medicine, Baltimore, MD, United States; e Department of Psychiatry and Behavioral Sciences, Johns Hopkins School of Medicine, Baltimore, MD, United States; f Department of Cognitive Science, Johns Hopkins University, Baltimore, MD, United States; g Department of Physical Medicine & Rehabilitation, Johns Hopkins University, Baltimore, MD, United States; h Department of Psychiatry and Behavioral Sciences, Johns Hopkins Medicine, Baltimore, MD, United States

**Keywords:** PPA, tDCS, Intervention, Spelling, Writing, Language rehabilitation, Prediction of treatment outcomes

## Abstract

The current study aims to determine the brain areas critical for response to anodal transcranial direct current stimulation (tDCS) in PPA. Anodal tDCS and sham were administered over the left inferior frontal gyrus (IFG), combined with written naming/spelling therapy. Thirty people with PPA were included in this study, and assessed immediately, 2 weeks, and 2 months post-therapy. We identified anatomical areas whose volumes significantly predicted the additional tDCS effects. For trained words, the volumes of the left Angular Gyrus and left Posterior Cingulate Cortex predicted the additional tDCS gain. For untrained words, the volumes of the left Middle Frontal Gyrus, left Supramarginal Gyrus, and right Posterior Cingulate Cortex predicted the additional tDCS gain. These findings show that areas involved in language, attention and working memory contribute to the maintenance and generalization of stimulation effects. The findings highlight that tDCS possibly affects areas anatomically or functionally connected to stimulation targets.

## 1. Background

Primary Progressive Aphasia (PPA) is a progressive loss of language abilities due to neurodegeneration (Mesulam, 1982, 2001, 2008). Language impairments in PPA may affect oral and written language production and comprehension, with the overall patterns of presentation typically classified into three variants: non-fluent variant PPA (nfvPPA), logopenic variant PPA (lvPPA), and semantic variant PPA (svPPA). Classification in one of these variants depends on deficits, but also neuroanatomical distribution of degeneration, and the underlying clinical pathology (Gorno-Tempini et al., 2011). Regarding anatomical distribution of degeneration in the brain, individuals with svPPA tend to have bilateral atrophy in the anterior temporal lobes, which is more severe in the left hemisphere (Gorno-Tempini et al., 2004, 2011; Mummery et al., 2000). The behavioral consequences of this atrophy include impaired word retrieval, object knowledge, and single-word comprehension in svPPA. Individuals with lvPPA have atrophy in the left inferior parietal lobe and the left posterior superior temporal gyrus (Gorno-Tempini et al., 2004; Josephs et al., 2013; Rohrer et al., 2010). These individuals tend to present with impaired word retrieval and sentence repetition with production of phonological paraphasias. Those with nfvPPA present with predominantly left frontal atrophy, including the left inferior frontal gyrus (IFG), pre-motor and supplementary motor areas, and the left insula (Grossman, Mickanin, Onishi, Hughes, D’Esposito, Ding, & Reivich, 1996; Josephs et al., 2006; Nestor et al., 2003). Individuals with nfvPPA present with non-fluent speech with agrammatic language production (possibly with apraxia of speech) and impaired syntactic comprehension of complex sentences.

Reading and spelling are impaired in specific ways in individuals of different variants, as explained in the next section. There are no disease-modifying treatments for PPA, but several behavioral (that is, language therapy; Beeson & Egnor, 2006; Henry et al., 2013; Meyer et al., 2013; Rapp & Glucroft, 2009) and neuromodulatory approaches (Cotelli et al., 2014; Gervits et al., 2016; Hung et al., 2017; Tsapkini, Frangakis, Gomez, Davis, & Hillis, 2014, 2018) have shown potential to reduce its consequences. The largest study to date using transcranial Direct Current Stimulation (tDCS) in PPA looked at the additional effects of tDCS over the left IFG in conjunction to written naming/spelling therapy (Tsapkini et al., 2018). The effects were encouraging: tDCS improved maintenance and generalization of therapy gains in relation to sham. However, not all individuals benefited equally from neuromodulation. Therefore, the question of which individuals benefit more from therapy and tDCS and how we predict which individuals will profit from neuromodulation remains unclear. In the current study, we aim to determine the relation between relative sparing or atrophy of specific brain areas (indexed by brain volumes) and response to a lexical retrieval intervention (written naming/spelling intervention) combined with transcranial Direct Current Stimulation (tDCS), compared to Sham. The results may help shape and individualize behavioral and stimulation protocols for individuals with different patterns of distribution of atrophy.

### 1.1. Spelling and PPA

Spelling impairments are prevalent across the three PPA variants, varying in presentation depending on which processing components are impaired (Neophytou, Themistocleous, Wiley, Tsapkini, & Rapp, 2019; Sepelyak et al., 2011; Shim, Hurley, Rogalski, & Mesulam, 2012). Correct spelling requires accurate processing in at least one of two processing routes. In the lexical route, a word is spelled by first recognizing the string of sounds that make up the heard word, identifying its meaning, and activating the knowledge of the orthography associated with spelling that particular concept that is stored in orthographic long-term memory. Alternatively, after hearing a string of speech sounds a listener can spell a word based on previous knowledge of language-specific regularities in correspondences between speech sounds and letters (a sublexical process known as phoneme-to-grapheme conversion, PGC). Importantly, this route can only produce correct spellings for regular words with predictable spellings (e.g., ‘bat’) or pseudowords that do not have an established spelling (e.g., ‘gum-prid’). Words with irregular spellings (e.g., ‘yacht’) must be spelled using an intact lexical-semantic route (Rapp & Fischer-Baum, 2015). Regardless of which route is used, the string of letters is stored temporarily in short-term memory to remain available as needed, for spelling in written or oral modalities (Caramazza, Miceli, Villa, & Romani, 1987). Individuals with nfvPPA tend to have difficulties with PGC at early stages of the disease, while those with svPPA tend to start by having poor lexical-semantic knowledge, and therefore attempt to spell using their intact PCG and produce incorrect, but phonologically plausible word spellings (Sepelyak et al., 2011; Shim et al., 2012).

Even though there is no disease modifying intervention for PPA, behavioral therapies have resulted in positive therapy outcomes. Patients with lvPPA improve in spelling treated words when treated using a spell-study-spell procedure (Rapp & Glucroft, 2009) and phoneme-to-grapheme conversion treatment (Tsapkini & Hillis, 2013a, 2013b). Another study by Meyer et al. (2013) reports therapy effects in terms of improvement in items that patients failed to name before therapy (remediation set) and maintenance of items in which patients had been successful at naming (prophylaxis set). Patients with both lvPPA and svPPA showed significant improvement for treated words in the remediation set, and those with svPPA showed better maintenance of trained words in the remediation set when compared to untrained words. Hence, the literature indicates that therapy leads to improvement for treated items, but there is little evidence of generalization of effects to untreated items. Studies using tDCS as a treatment adjuvant have yielded more promising results in terms of generalization.

### 1.2. Transcranial direct current stimulation and language therapy

Neuromodulation techniques have been combined with behavioral approaches to enhance language performance in a variety of clinical and non-clinical populations. In particular, tDCS is a neuromodulation technique that can have an effect on the excitability of the stimulated brain areas (Kuo, Polanía, & Nitsche, 2016). A weak electrical current is administered through the scalp, targeting brain areas functionally associated with the cognitive functions to enhance. Previous literature shows that tDCS can enhance language performance in healthy individuals (e.g., Flöel, Rösser, Michka, Knecht, & Breitenstein, 2008), albeit some studies report null results (e.g., Westwood, Olson, Miall, Nappo, & Romani, 2017). In post-stroke aphasia, greater response to behavioral interventions has also been reported for individuals receiving tDCS, compared to Sham (de Aguiar, Paolazzi, & Miceli, 2015; Fridriksson et al., 2018).

In PPA, tDCS paired with oral picture naming therapy results in greater improvement than Sham, for trained (Cotelli et al., 2014) and untrained items (Roncero et al., 2017), with maintenance of therapy gains reported up to 12 weeks (Cotelli et al., 2014). Other studies did not include a Sham control, but also showed that tDCS paired with modified semantic feature analysis (Hung et al., 2017) and narrative therapy (Gervits et al., 2016) result in statistically significant improvement relative to baseline performance.

In the written language domain, Tsapkini et al. (2014) compared anodal tDCS administered to the left IFG with Sham, both combined with 15 sessions of spelling therapy. Spelling intervention consisted of a combination of training of phoneme-to-grapheme conversion and written naming therapy. Post-therapy improvement in spelling trained items was detected both for sham and tDCS phases. Improvement was greater in tDCS when compared to Sham for untrained items immediately, two weeks, and two months after therapy, and for trained items two months after therapy (but not immediately after or two weeks after therapy). These findings were replicated in 36 participants in the largest randomized sham-controlled clinical trial in PPA to date (Tsapkini et al., 2018). In the functional connectivity analysis of these tDCS effects, tDCS-related improvement correlated with changes in functional connectivity between the stimulated area (the left frontal lobe targeting the IFG) and other temporal language areas. In particular, tDCS modulated the connectivity between the left IFG triangularis and the left Inferior Temporal Gyrus and between the left IFG orbitalis and the left Middle Temporal Gyrus (Ficek et al., 2018). Understanding the neural correlates of therapy and tDCS-mediated change may lead us to better targeting of interventions to cause generalization and potentially functional communication changes. In as much as it is important to define the neural mechanisms of tDCS, it is equally important to be able to determine which brain areas are needed to respond to neuromodulation and how atrophy may affect therapy effects.

### 1.3. Neuroanatomical predictors of language recovery and intervention(s)

The current literature on anatomical correlates of response to language interventions or language recovery is mostly based on studies with individuals with post-stroke aphasia, not involving neuromodulation. From a behavioral standpoint, Lazar et al. (2010) have shown the amount of that aphasia recovery is proportional to initial severity: patients tend to improve to about 70% of their maximal potential recovery. Several studies also attempted to identify anatomical predictors of recovery, mapping the ‘natural history’ of recovery after stroke. For instance, Saur et al. (2010) used a machine learning approach and found that baseline severity and age combined with activation of the right frontal region (largely encompassing the IFG) distinguished patients with good vs. bad recovery with 86% accuracy. Hope et al. (2017) report that oral and written naming changes after stroke are related to structural changes in the right temporal lobe and pre-central gyrus. At the subcortical level, Forkel et al. (2014) found a correlation between the volume of the long segment of the right arcuate fasciculus and decrease in post-stroke aphasia severity across a 6-month period. Similar results were reported by Wang, Marchina, Norton, Wan, and Schlaug (2013) and Hillis et al. (2018), for acute stroke. A more recent approach focuses on functional anomaly maps, rather than structural data alone, having the potential to measure the relation between improvement and dysfunction related to a lesion, but in distant areas (DeMarco & Turkeltaub, 2018).

Others identified neuroanatomical and neurofunctional predictors in the context of a behavioral intervention study. Meinzer et al. (2010) found that greater improvements in naming treated items after constrained induced (intensive) language therapy could be predicted by the integrity of the hippocampus and adjacent white matter. van Hees et al. (2014) found that improvement in naming words treated using phonemic cues was predicted by right middle-temporal gyrus amplitude of low-frequency fluctuations in resting state fMRI scans collected before therapy. Improvement in naming untreated items after semantic and phonemic cueing therapy was correlated with overall integrity of brain functional connectivity and connectivity within the left temporal lobe (Bonilha, Gleichgerrcht, Nesland, Rorden, & Fridriksson, 2016).

In individuals with non-fluent variant primary progressive aphasia, improvements in oral naming of treated nouns were correlated with grey matter volume in the left fusiform, left middle temporal, and right inferior temporal gyri (Cotelli et al., 2016). The same study reported that improvement in untreated verbs correlated with grey matter volume in the left middle temporal gyrus. Treatment was restricted to nouns and consisted of repetition and reading of the target, each followed by articulatory suppression and then by an attempt at naming the target. Meyer et al. (2013) also studied the relation between baseline brain volumes and naming scores obtained after therapy using orthographic and phonological cues to facilitate picture naming. They found that individuals with smaller volumes of the left temporal pole presented lower post-therapy accuracy for untrained prophylaxis items (which were named correctly before therapy), indicating more rapid decline in individuals with smaller baseline volumes, or lesser potential for generalization. In addition, greater volumes of the left inferior temporal gyrus were positively correlated with post-therapy accuracy for untrained remediation items (that is, those named incorrectly before therapy), further supporting the authors’ claim that greater potential for generalization may be present in individuals with preserved inferior and anterior temporal areas.

A previous study provided evidence that individual anatomical differences may lead to different current distributions and therefore to differential response to tDCS (Kim et al., 2014). Furthermore, McConathey et al. (2017) report that baseline performance predicts effects of tDCS in PPA. While baseline performance may be related to overall severity of language impairments or overall severity of degeneration, in the current study we test the hypothesis that brain volumes in the stimulated region and in networks relevant for written naming, spelling and learning may also lead to differential responses to stimulation. Identification of areas associated with improvement after combined spelling and tDCS intervention in individuals with PPA will allow for a better individual tailoring of stimulation in terms of brain areas to stimulate.

## 2. Method

### 2.1. Participants

**Fig. 1.**
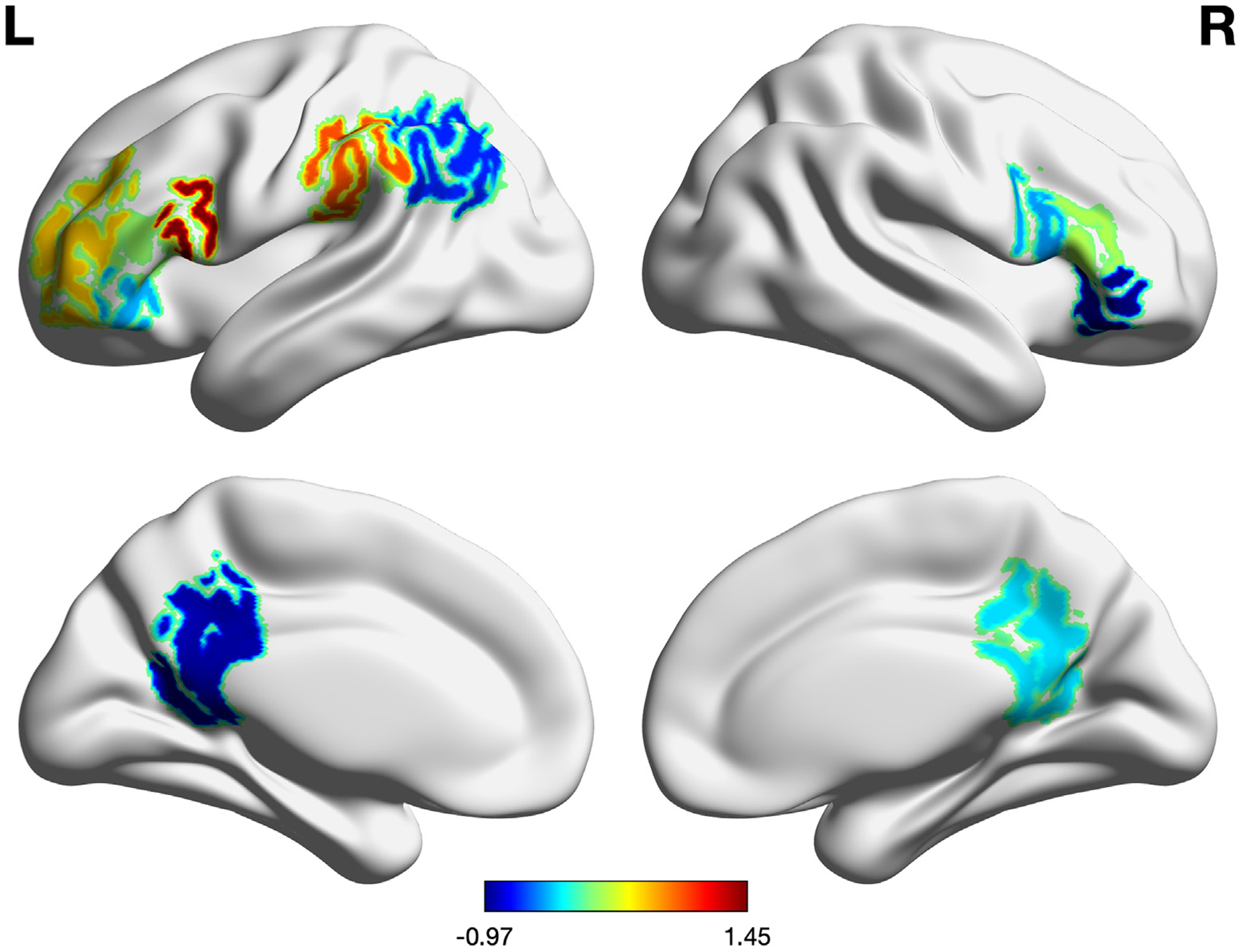
t-scores brain volume comparison between the tDCS and Sham group of participants.

Thirty individuals with PPA were included in this study (15 males). The sample included 9 individuals diagnosed as nfvPPA, 14 as lvPPA, and 7 as svPPA. Patients were on average 66.4 (± 6.7) years old and reported being at 4.7 (± 3.0) years post-onset of symptoms. Language abilities were mild to moderately impaired at the time of enrollment, as measured by the FTDCDR language item (1.9 ± 0.8) (Knopman et al., 2008). The FTDCDR sum across all items was of 7.5(± 4.8) (see Table 1 for demographic and descriptive statistics). Patients were randomized to either the tDCS or Sham group with equal probability, so systematic biases in baseline variables are unlikely. In any case, we compared the distribution of the tDCS and Sham samples and found no significant differences in type of PPA variant (*Fisheŕs exact test*, p = 0.90), language severity in the language item of the FTLD-CDR (t(27.73) = 0.51, p = 0.62), and pre-therapy scores in the outcome measure for trained and untrained items (trained: t(27.64) = 0.47, p = 0.64; untrained: t(27.92) = 1.35, p = 0.19). Furthermore, the tDCS and Sham groups did not differ in the volume of their left and right IFG, or of any of the regions of interest selected by the prediction model (p > 0.15 in all comparisons) (see Fig. 1, and Sections 3.2 and 3.3). Descriptive statistics for each region are reported as Supplementary Materials.

### 2.2. Design of treatment protocol

**Fig. 2.**
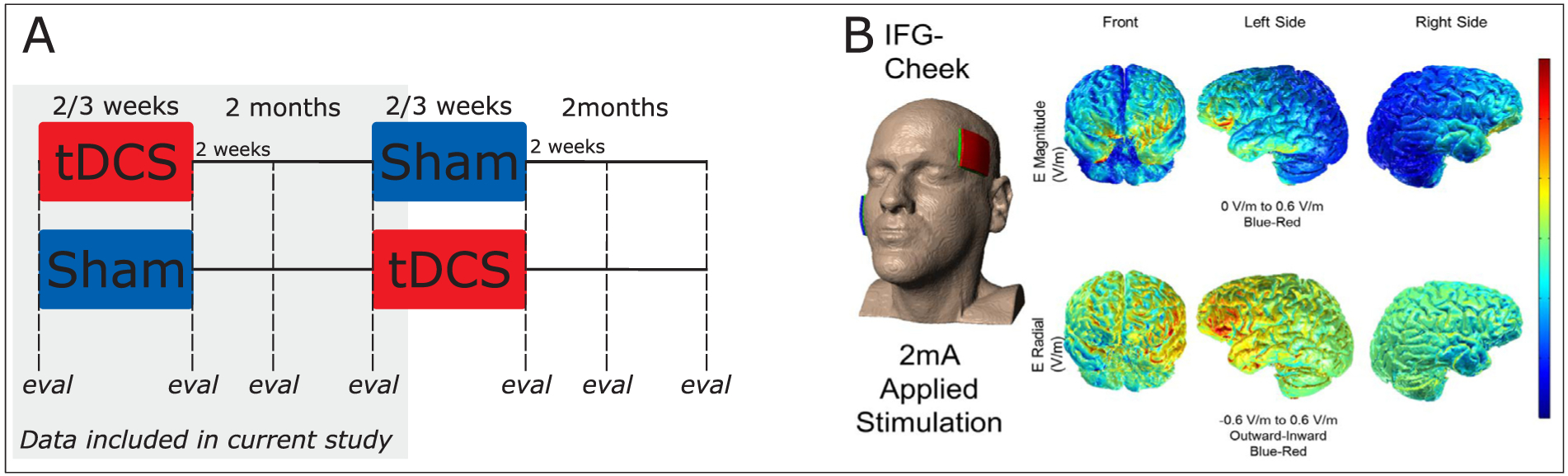
Study design and model of current distribution for stimulation to the IFG. Panel A: study design. The grey-shaded area corresponds to the data included in the present study. Panel B: model of current distribution for used stimulation montage (image courtesy: Dr. Marom Bikson).

The data reported in this study are part of a larger clinical trial (ClinicalTrials.gov; Identifier: NCT02606422; Tsapkini et al., 2018). Individuals with PPA are trained on written naming/spelling abilities over two phases, separated by a washout period of 2 months (see Fig. 2, panel A). Each therapy phase included 10–15 therapy sessions (depending on individual availability). Participants received the same behavioral therapy across phases but were randomized to receive either anodal tDCS or Sham in the first phase, and the opposite stimulation condition in the second phase. The current study focuses on the first therapy phase for all participants, in a between-subjects design.

#### 2.2.1. tDCS

Anodal tDCS (or Sham) was delivered in the first 20 min of each 45-min therapy session, using two 5 × 5 cm electrodes, with the Soterix 1 × 1 Clinical Trials device. The anode targeted the left IFG, and was therefore placed over the F7 co-ordinate of the 10–20 system (Homan, 1988). Furthermore, the accuracy of the pairing of the IFG with the scalp co-ordinate was checked by co-registering this landmark with MRI data using a fiducial marker, separately for each individual. The cathode was placed over the right cheek. A model of current distribution for this montage is presented in Fig. 2, panel B. Sham was delivered by ramping the current up and down in the first and last 30 s of the 20-min stimulation period (Gandiga, Hummel, & Cohen, 2006). The IFG was chosen for stimulation based on its engagement in multiple language processes, including in lexical and sublexical spelling mechanisms. It is engaged in active retrieval (Petrides, Alivisatos, & Evans, 1995) in general, and in particular in semantic selection (Thompson-Schill, D’Esposito, & Kan, 1999), in orthographic long-term memory (Purcell, Turkeltaub, Eden, & Rapp, 2011 for a meta-analysis), and phoneme-to-grapheme conversion (Rapcsak et al., 2009; see Tsapkini & Hillis, 2013a, 2013b, for a review).

#### 2.2.2. Behavioral treatment

Behavioral therapy consisted of oral and written naming + spelling or spelling-only intervention using the spell-study-spell procedure (modified from Beeson & Egnor, 2006, CART, Copy and Recall Treatment, for PPA; Rapp & Glucroft, 2009). For each trained item, the treatment task followed the following steps:

1. The participant was presented with a picture and asked to orally name the item.
2. If the participant made errors during oral naming, s/he received semantic (e.g., something sharp used for cutting paper, for *scissors*) and phonemic cues to facilitate naming.
3. The spell-study-spell procedure was conducted. That is, the participant first attempted to write the target. If correct, s/he was asked to inspect and copy the written word three times. If incorrect, the participant was prompted with semantic cues, then s/he was provided with the correct spelling of the target word, then read the word, named each of the letters, and copied the word 5 times.

Ten individuals received a modified version of the treatment where no pictures were presented. This means that steps (1) and (2) was not conducted for those individuals. However, in all cases, treatment required participants to retrieve orthographic word forms from long-term memory and entailed semantic cueing to facilitate performance when needed. Semantic cues provided for spoken or written responses were identical. Given the central nature of the semantic system in language processing (e.g., Coltheart, Rastle, Perry, Langdon, & Ziegler, 2001; Patterson & Shewell, 1987; Whitworth, Webster, & Howard, 2014) we provided identical semantic cues in order to facilitate oral or written responses. These cues are thought to facilitate semantic processing precedes the selection of the oral or written output modality.

### 2.3. Imaging data collection and processing

Structural MRI scans were acquired using a 3 T Phillips Achieva MRI scanner using a 32-channel head coil, at the Kennedy Krieger Institute at Johns Hopkins University. Scans were acquired axially using a T1-weighted MPRAGE sequence, with a scan time of 6 min (150 slices), with 3D inversion recovery, magnetization-prepared rapid gradient, isotropic, and voxels with 1 × 1 × 1 mm^3^ resolution. The field of view (FOV) was of 224 × 224 mm^2^, TR/TE of 8.1/3.7 ms, flip angle of 8 degrees, and SENSE acceleration factor of 2.

Brain volumes were extracted from anatomical scans using MRIcloud, a cloud-based platform that performs automated image parcellations using an atlas-based analysis. Each individual’s scan was initially parcellated into 283 regions of interest (ROIs) (Mori et al., 2016) using a multi-atlas fusion label algorithm and large deformation diffeomorfic metric mapping (Ceritoglu et al., 2013; Miller, Beg, Ceritoglu, & Stark, 2005; Tang et al., 2013). The combination of a highly accurate diffeomorphic algorithm with the use of multiple atlases minimizes inaccuracies in mapping and segmenting ROIs. For the purposes of this study, and to better measure relative regional atrophy, the volume of each ROI was normalized by the total intracerebral volume, which was calculated as the total brain tissue, minus the myelencephalon and cerebrospinal fluid (e.g., Ficek et al., 2018). Regions of interest were then reduced to 21 left hemisphere areas and their right hemisphere homologues. ROI selection depended on a region’s theoretical relevance for spelling and/or for learning. Furthermore, left-right asymmetry (left hemisphere volume minus right hemisphere volume) and global atrophy (intracranial volume^4^ divided by intracerebral volume) were also calculated and included in the analyses as predictors so that significance of any brain volume would be adjusted for overall atrophy as described in Section 2.5.

### 2.4. Outcome measures

Before, immediately after, 2 weeks after, and 2 months after therapy, participants completed written picture naming and spelling to dictation tasks and were tested with trained and untrained items so that generalization of effects could be detected. Words chosen for the trained and untrained sets were matched in both length and frequency, based on the norms from the MRC psycholinguistic database (Coltheart, 1981). In the written naming task, participants were shown a picture of the item to be named and were asked to write the target word. In the spelling to dictation task, participants heard the target word and attempted to write it. Participants who received the modified version of therapy (without steps (1) and (2) described above) were assessed using the spelling task. Regardless of the variation of the task administered, the primary outcome measure used to detect therapy related changes in performance was letter accuracy. This way, all written responses were scored for the percentage of correct letters. The scoring system evaluates the accuracy for each letter taking into account errors of letter deletion, addition, substitution, and movement (Goodman & Caramazza, 1985). For each follow-up time-point, we calculated the percent change *Y*_f_ in correctly written letters by subtracting the pre-therapy letter accuracy (percentage of correctly spelled letters) from each post-therapy letter accuracy (f = immediately after therapy, 2 weeks, and 2 months after therapy).

### 2.5. Analyses

#### 2.5.1. Establishing the effect of tDCS

Effects of tDCS were established based on the change in letter accuracy immediately after sham minus before sham, 2 weeks after sham minus before sham, and 2 months after sham minus before sham; and the analogous changes under tDCS. Estimates of these effects, standard errors, and confidence intervals were obtained using the generalized estimating equation method with robust estimation of the variance of the estimates (Liang & Zeger, 1986). This robust method accounts for the possible correlation among the repeated outcomes across times within an individual (Liang & Zeger, 1986). *P-*values are exact (non-parametric). Additional details of this analysis are reported in Tsapkini et al. (2018).

#### 2.5.2. Identifying predictors of tDCS effects

In this study, our aim is to identify brain regions that the volume (or the level of atrophy) can predict the tDCS effects on the language behavior outcome^5^. Therefore, we considered the forward feature selection approach to first identify the brain regions that made a significant contribution in predicting the tDCS effect. After identifying brain regions, we fitted a multiple linear regression model to further elaborate the relationship. However, the number of brain regions is greater than the number of subjects. Thus, fitting a multiple linear regression with all brain regions is not reliable and a feature selection step is required. We added details about the analysis below, and in the supplementary material.

We evaluate how baseline volumetric data *V* may be modifying any effect of treatment stimulation *T* (tDCS vs sham) on the primary outcome measure *Y*_*f*_ (for each follow-up f) as defined above. The modifying effect of each variable (be it an anatomical region of interest) is measured after adjusting for pre-therapy letter accuracy *Y*_*pre*_and Global Atrophy *GA*. To do this, we fitted the model

$$E\left(Y_{f}|T=\text{tDCS},V,Y_{\text{pre}},GA\right)-E\left(Y_{f}|T=\text{Sham},V,Y_{\text{pre}},GA\right)$$

$$\alpha_{0f}+\alpha_{1f}Y_{\text{pre}}+\alpha_{2f}GA+\beta_{1f}V_{1}+\cdots +\beta_{\text{max},f}V_{\text{max}},$$

where α, and β’s are model coefficients to be estimated. This model can be fitted without specifying *E*(*Y*_*f*_ |*T* = *Sham*,*Y*_*pre*_, *GA*), using the approach of Tian, Alizadeh, Gentles, and Tibshirani (2014), which is also equivalent to the fitting of structural nested models (Vansteelandt & Joffe, 2014). Because not all the above predictors can be fitted at the same time, we used the following forward stepwise selection strategy (i) we forced fitting of the factors *Y*_*pre*_, *GA*, throughout; (ii) from the remaining factors, we select the one that provides the largest increment in the cross-validated coefficient of determination (ΔR^2^); if this increment is significantly greater than 0, we include that factor in the model. We repeated (ii) until no remaining factor provided ΔR^2^ significantly greater than 0. Hence, factors that are significantly associated with treatment response are predictors of the added effect of left IFG tDCS when compared to Sham. For an ROI selected in the model, we also report the coefficient β standardized to be the change in the tDCS vs Sham effect (as a fraction of its standard deviation) that is associated with one standard deviation change in the ROI volume.

We also considered various approaches including (a) without imposing fitting factors *Y*_*pre*_, *GA* but including them as well as language severity and the number of graphemes for model selection; (b) normalizing brain volumes by the contralateral volume. The results remain basically the same, therefore, in the next section, we only present the findings from the above-mentioned analysis strategy.

## 3. Results

### 3.1. Effects of tDCS

**Fig. 3.**
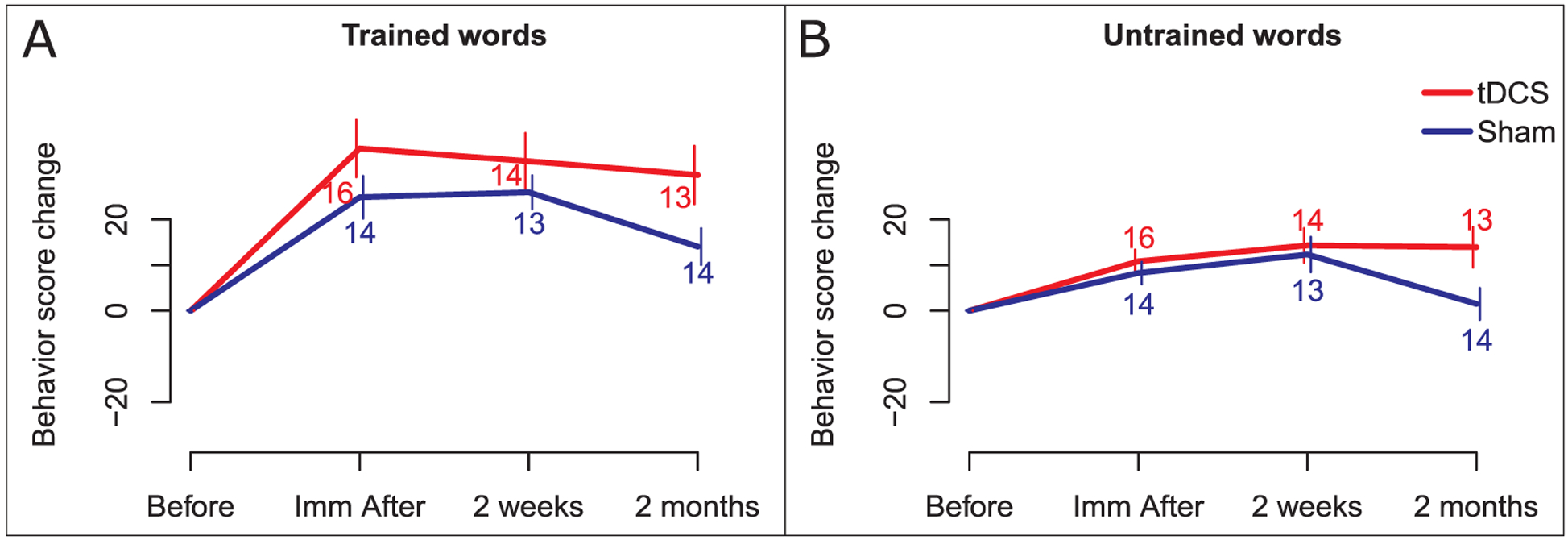
Effects of tDCS on behavioral score change. Change from baseline on behavioral scores is presented in the y axis, and each post-therapy time-point is presented on the x-axis. Scores are presented in red for the tDCS group and in blue for the Sham group. Panel A: tDCS effect for trained words. Panel B: tDCS effects for untrained words.

For trained words, there was no significant effect of tDCS at the group level on change in letter accuracy immediately or 2 weeks after therapy (immediately after therapy: additional gain, 10.7; SE, 7.8; p = 0.18; 2 weeks after therapy: additional gain, 6.8; SE, 7.2; p = 0.395). A significant effect was found at 2 months after therapy (additional gain for tDCS over sham, 15.7; standard error (SE), 7.5; p = 0.04). For untrained words, effects of tDCS were marginally significant at immediately after therapy (immediately after therapy: additional gain, 3.8; SE, 2.2; p = 0.065), non-significant at two weeks after therapy (additional gain, 2.0; SE, 5.4; p = 0.705), and significant 2 months after therapy (additional gain, 12.4; SE, 5.6, p = 0.04) (see Fig. 3, panels A and B for trained and untrained words, respectively).

### 3.2. Predictors of the stimulation effect for trained words

**Fig. 4.**
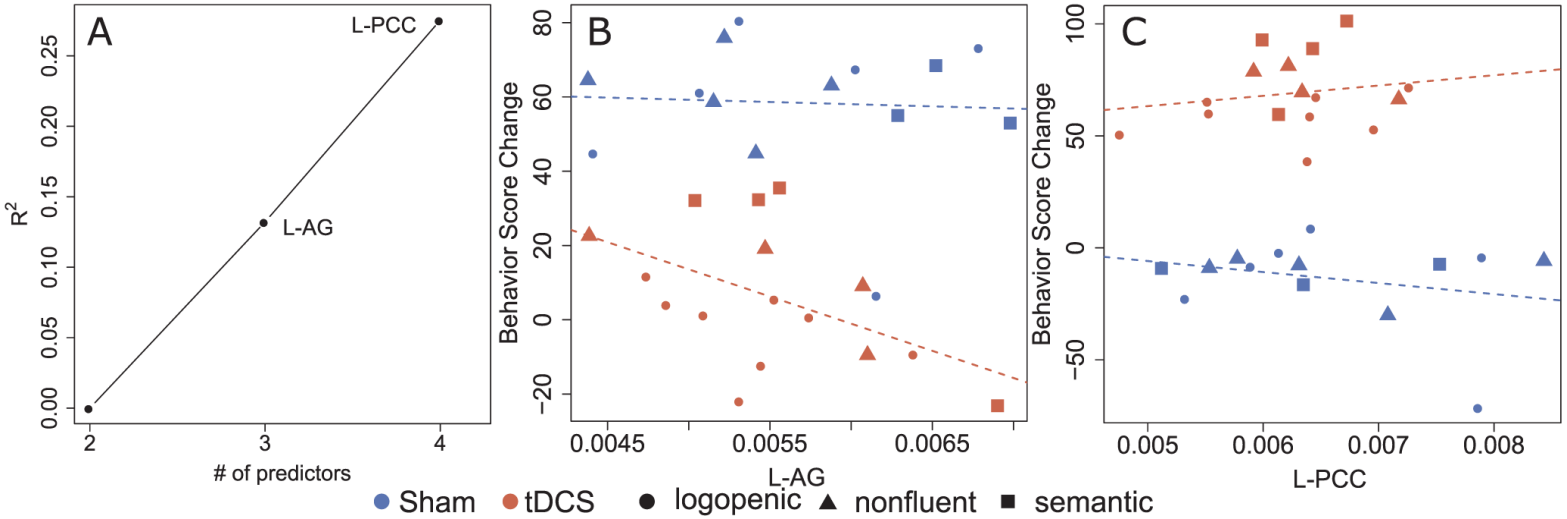
Predictors of percent change in spelling accuracy: trained words (immediately after therapy). Brain regions showing significant interactions between stimulation and volumetric data in predicting change in letter accuracy are presented. Panel A represents R^2^ increase obtained when adding each ROI to the model containing adjustments for pre-therapy scores and Global Atrophy. Panels B and C contain scatter plots of behavioral score (that is, % letter accuracy) change in relation to the pre-therapy assessment (on the y axis) versus brain volume. In each scatter plot, the y axis is the behavioral score change adjusted using regression coefficients, that is, accounting for the remaining variables included in the model. Red represents points for patients in the tDCS group, and blue for the Sham group. Participants with different PPA variants are represented with different symbols: ● lvPPA; ▲ nfvPPA; ■ svPPA.

Immediately after therapy, effects of tDCS on change in letter accuracy for trained words were associated with volumes of the left Angular Gyrus (l-AG; ΔR^2^ = 13.2%, β = −0.51 p < 0.01). As illustrated in Fig. 4 (panel B), smaller volumes of the left AG were associated with greater benefit from tDCS than larger volumes, while there is no association in the Sham condition. After controlling for left AG volumes, the left Posterior Cingulate Cortex (l-PCC) volumes were also associated with stimulation effects (ΔR^2^ = 14.3%, β = 0.40, p < 0.05). Therapy response under tDCS becomes relatively greater than for individuals receiving Sham as the left PCC volume increases, while the pattern is in the opposite direction for Sham (Fig. 4, panel C; results are summarized on Table 2). No brain regions predicted stimulation effects at 2 weeks or 2 months after therapy, independently from the initial performance and overall atrophy.

### 3.3. Predictors of the stimulation effect for untrained words

**Fig. 5.**
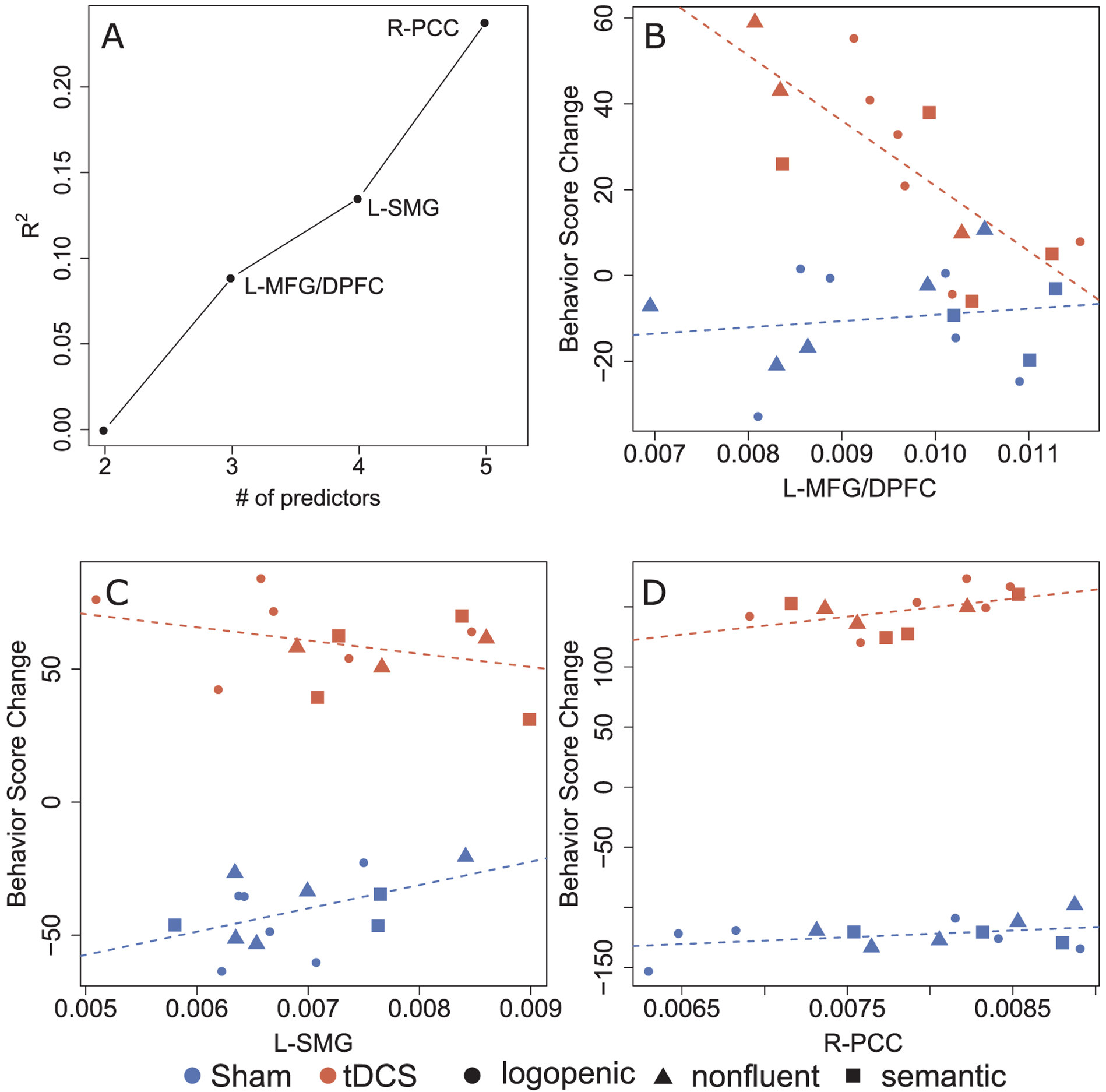
Predictors of percent change in spelling accuracy: untrained words (2 months after therapy). Brain regions showing significant interactions between stimulation and volumetric data in predicting change in letter accuracy are presented. Panel A represents R2 increase obtained when adding each ROI to the model containing adjustments for pre-therapy scores and Global Atrophy. Panels B to D contain scatter plots of behavioral score (that is, % letter accuracy) change in relation to the pre-therapy assessment (on the y axis) versus brain volume. In each scatter plot, the y axis is the behavioral score change adjusted using regression coefficients, that is, accounting for the remaining variables included in the model. Red represents points for patients in the tDCS group, and blue for the Sham group. Participants with different PPA variants are represented with different symbols: ● lvPPA; ▲ nfvPPA; ■ svPPA.

For changes immediately and two weeks after therapy, adding volumetric data to the baseline model did not improve the R^2^ of the model. However, two months after therapy the effects of tDCS on change in letter accuracy for untrained words were related to the volume of the left Middle Frontal Gyrus/Dorsal Prefrontal Cortex (l-MFG/DPFC; ΔR^2^ = 8.9%, β = −0.97, p < 0.001). Individuals with smaller left MFG volumes benefited more from treatment if they received tDCS, compared to those receiving Sham (Fig. 5, panel B). After controlling for left MFG volumes, the Supramarginal Gyrus (l-SMG) predicted stimulation effects (ΔR^2^ = 4.6%, β = −0.39, p < 0.05). Here too, smaller volumes were associated with better response to treatment in the tDCS condition compared to Sham (Fig. 5, panel C). Furthermore, the right posterior Cingulate Cortex (PCC; ΔR^2^ = 10.3%, β = 0.38, p < 0.05) predicted stimulation effects when volumes of the two other ROIs were accounted for. In this case, larger right PCC volumes were associated with greater benefit from therapy paired with tDCS, compared to Sham (Fig. 5, panel D).

## 4. Discussion

In the present study we have identified predictors of the response to tDCS over the left IFG for trained and untrained words in written naming/spelling, after adjusting for pre-therapy scores and global atrophy. For trained words, we detected significant predictors only for immediate stimulation effects, that is, those measured immediately after therapy. However, for untrained words, we detected predictors of long-term stimulation effects, specifically, two months after therapy. For trained words, greater benefit from stimulation was associated with smaller volumes of the left AG, and larger left PCC volumes. For untrained words, greater benefit of stimulation was associated with smaller left MFG volumes, smaller left SMG volumes, and larger right PCC volumes.

With regard to behavioral changes, tDCS had significant effects on changes of letter accuracy for both trained and untrained items, particularly at two months after the end of intervention. Hence, stimulation effectively increased the maintenance of therapy effects. Even in the absence of group effects of tDCS for some time-points for either trained or untrained words, asking whether certain patterns of atrophy may be associated with greater response may help to elucidate reasons behind null effects at the group level. The Discussion will focus on the relation between such patterns of atrophy and stimulation effects. For a Discussion of tDCS effects detected in this clinical trial, we refer the reader to Tsapkini et al. (2018).

### 4.1. Task-relevant volumetric predictors

For several left-hemisphere regions (AG, MFG, SMG), greater benefit for stimulation was associated with smaller brain volumes. A simple explanation would be that more severe degeneration in these areas is equivalent to more severe behavioral deficits, and therefore individuals with PPA with greater atrophy have a greater potential to show significant change. Firstly, we will acknowledge that although the relationship between volumetric reduction and cortical atrophy is intuitive, it may be a confounder, from the technical point of view. Differences in MRI contrast lead to shifts in the gray-white matter boundary as observed in the T1-weighted images, which reflect in increases or decreases of the measured cortical volume. The image contrasts may be affected by uncontrollable artifactual factors (e.g., scan variability) and biological factors (e.g., white matter composition, vascular status). Therefore, the cortical volumetric reduction not necessarily indicates atrophy, but a composition of phenomena that are shifting the gray-white matter boundary. This volume-intensity coupling, a well-known problem in image analysis, certainly makes the intuitive interpretation of our findings harder. Having said that, the intuitive explanation relating volumetric reduction to cortical atrophy is still likely, as the neuroimaging signal can be thought of as an important correlational marker of atrophy.

Global Atrophy and pre-therapy scores in the outcome measure were both included as covariates in all models, hence controlling for general effects of severity. An opposite direction of relation between atrophy and effects of behavioral therapy was described by Meyer et al. (2013), who report that individuals with lower volumes of the left temporal pole presented lower post-therapy accuracy for untrained prophylaxis items (which were named correctly before therapy). This was interpreted as indicating more rapid decline in individuals with smaller baseline volumes, or lesser potential for generalization. Given the negative correlations that we report (that is, smaller volumes associated with greater improvement) we cannot state, like Meyer and colleagues, that greater degeneration equates reduced potential for change. We should also add that the behavioral results of the intervention protocol used in the current study show that participants, as a group, showed gains in letter accuracy and sustained them for both tDCS and sham conditions (Tsapkini et al., 2018).

Stimulation may induce a more functional activity pattern in these regions, leading to behavioral improvement. This interpretation is in line with the report by (Ficek et al., 2018), that tDCS-related improvement correlates with changes in functional connectivity between the stimulated area and other task-relevant areas. We should consider, however, why a region with smaller volume would be more susceptible to these functional changes towards a more functional activity pattern. This may be linked again to the severity of such dysfunction; that is, regions with greater atrophy may be less functional and therefore show a greater potential for functional improvement. While we included Global Atrophy and baseline scores as general markers of severity, these may not account for regionally specific structural and functional degeneration. Hence, the type of severity predicting stimulation effects may be that of the local structural atrophy and dysfunction, rather than the overall atrophy and general spelling impairment, which are accounted by the baseline variables in the model.

Regardless of the exact mechanism of change, the regions identified are structurally connected to the stimulated area, and play important roles in spelling, as discussed next. Ultimately, this leads to the need to consider structural connectivity and network relevance when deciding on stimulation targets. Both the Angular Gyrus and the Supramarginal Gyrus are structurally connected the stimulated IFG via the superior longitudinal fasciculus (SLF-III; Frey, Campbell, Pike, & Petrides, 2008; Seghier, 2013). Furthermore, given the large size of the stimulation electrode use, and the current distribution shown on Fig. 2, it is likely that the middle frontal gyrus also received stimulation. In any case, this region is connected to the IFG via short association U-shaped fibers (Barredo, Verstynen, & Badre, 2016). Hence, even though the amount of atrophy in the IFG did not predict effects of stimulation, the integrity of the regions connected to the left IFG does. Importantly, apart from being anatomically connected to the stimulated area (left IFG) the AG, SMG, and MFG play important roles in processing written language. These results align with the behavioral results reported by Tsapkini et al. (2018) that individuals with nvfPPA, who show atrophy in frontal and fronto-parietal regions, show greater tDCS effects.

The AG is thought to be involved in mapping visual stimuli to linguistic representations, showing functional connectivity during reading with extrastriate and posterior temporal areas, in healthy but not dyslexic adults (Horwitz, Rumsey, & Donohue, 1998). It has also been causally linked to semantic processing (Price, Peelle, Bonner, Grossman, & Hamilton, 2016). The left supramarginal gyrus has been linked to converting orthographic information to phonology (letter to sound correspondences) when reading words in languages with trans-parent vs. opaque orthography (Law et al., 1991; Price, 1998). While the middle frontal gyrus is typically associated with cognitive control (in particular, inhibition) and visual attention (Giesbrecht, Woldorff, Song, & Mangun, 2003; Hopfinger, Buonocore, & Mangun, 2000), it has also been shown to be active during both word and nonword reading (Borowsky et al., 2006), therefore potentially supporting attentional demands of reading. It is important to note that, this region has recently been associated with spelling and in particular the orthographic working memory (Rapp, Purcell, Hillis, Capasso, & Miceli, 2015).

We should also briefly discuss why the left AG predicts the effect of stimulation for trained words, while the other two regions relate to effects of stimulation for untrained words. This may relate to the hypothesized role of these areas in spelling. As stated above, the left AG may be involved in linking specific orthographic representations to semantics (Horwitz et al., 1998). Hence, potential learning facilitated by enhanced function in this area would be specific to orthographic representations for which there was exposure during training (the trained words). Differently, the left supramarginal gyrus has been linked to letter to sound correspondences (Price, 1998), which consist of a set of rules which are applied to many words. In fact, in post-stroke aphasia, generalization in spelling is most often found in studies treating phoneme-to-grapheme correspondences (Luzzatti, Colombo, Frustaci, & Vitolo, 2000; de Partz, Seron, & der Linden, 1992). Finally, the attentional demands of reading associated with the left MFG (Borowsky et al., 2006) are also relevant for trained and untrained words. Hence, stimulation induced enhancements in functioning of these last two areas may have an impact in spelling untrained words.

An implication of the finding that individuals with smaller left AG, SMG, and MFG areas show greater benefit if receiving tDCS is that these areas may also be effective targets for stimulation, even in individuals with greater degeneration. However, it is also possible that stimulation to the left IFG was effective (as reported by Tsapkini et al., 2018) precisely because of its central role in a network, linking areas with different, but important roles in spelling. Hence, stimulating any individual node of that network may not be as effective. Future research may explore differential effects of stimulation to nodes and hubs in functional networks.

### 4.2. The posterior Cingulate Cortex and its contribution to stimulation effects

Left and right posterior cingulate were associated with stimulation effects, for trained and untrained words, respectively. The PCC has been associated with episodic memory retrieval (Daselaar, Veltman, Rombouts, Raaijmakers, & Jonker, 2003; Konishi, Wheeler, Donaldson, & Buckner, 2000), with lesion to this region resulting in episodic memory impairment (Valenstein et al., 1987). Other studies also indicate that the PCC is involved in working memory (Hampson, Driesen, Skudlarski, Gore, & Constable, 2006) in regulating the focus of attention (for a review see Leech & Sharp, 2014). Given its role in memory and attention, it is thought to support learning (Pearson, Heilbronner, Barack, Hayden, & Platt, 2011). It is also an area that is very susceptible to degeneration, showing early accumulation of Alzheimer’s disease pathology, and reduced metabolic rate in very early Alzheimer’s Disease (Minoshima et al., 2004).

Why should greater benefit to tDCS then be related to larger PCC volumes? Given the contribution of the PCC to attention, it is possible that individuals with greater volumes in this area simply have a greater ability to attend to therapy stimuli and procedures and, consequently, a greater potential learn. If tDCS can enhance the ability to learn spelling of trained and untrained words, it is logical that this happens to a greater extent in individuals with a greater baseline attention. Importantly, learning requires attention, both for trained and untrained words.

### 4.3. Limitations

Tsapkini et al. (2018) report differences in response to stimulation across PPA variants, with patients with semantic variant PPA showing no additional benefit under tDCS compared to Sham. This may be due to the characteristics of stimulation (stimulation site, polarity, intensity, modality; de Aguiar et al., 2015). However, not only were there limited tDCS effects, but there was also limited generalization in this group. Hence, the lack of a stimulation effect, particularly for untrained words, can also be an indication that the treatment provided is not suitable to yield generalization in individuals with semantic variant PPA, and this way, generalization cannot be enhanced by tDCS. Thus, neuroanatomical predictors of stimulation effects in svPPA should be identified in the context of a behavioral treatment more likely to yield substantial change in behavioral performance, in particular for untrained words. Unfortunately, improvement in spelling of untrained words in svPPA has not been reported in previous treatment studies.

Another limitation of the current study is that there may be different reasons why we find a relation between brain volumes and response to tDCS. We discuss that areas functionally relevant for spelling were selected as predictors, which implies that these regions’ functional role is important for obtaining behavioral improvement. However, a relation between the volume of regions connected to the left IFG and the effect of tDCS may also indicate that the volumes of those regions affect current distribution. In this second explanation, the functional role of each region is irrelevant. However, the fact that regions identified are involved in cognitive functions relevant for spelling is unlikely to be a coincidence. Furthermore, the study of Ficek et al. (2018), reports on changes in resting state MRI data in participants of this same clinical trial. They detect tDCS-related changes in functional connectivity between the left IFG triangularis and orbitalis and the left inferior temporal and middle temporal gyri, respectively. Hence, it is likely that the functional role of regions connected to the stimulated brain areas is relevant to and effected by stimulation effects.

## 5. Conclusion

Greater left IFG tDCS effects were associated with smaller volumes of several areas that are known to have specific contributions to spelling, including in mapping orthographic to semantic representations (left AG), letter to sound correspondences (left SMG), and controlling the attentional demands of spelling (left MFG). In addition, the role of the left and right PCC volumes may suggest that individuals with a greater baseline attention may be at an advantage to benefit from training effects enhanced by stimulation. These findings provide guidance towards the use of tDCS in individuals with PPA, stressing the importance of considering the function of the areas structurally connected to stimulation targets. Future research may inquire whether the above-mentioned areas can be suitable stimulation targets to pair with behavioral interventions for spelling in PPA.

## 6. Statement of significance

The present study is highly relevant to the mission of the journal because it seeks to inform about the relationship between brain and language function. It aims to identify brain areas that predict electrical stimulation effects on language therapy and, in particular, written naming and spelling.

## Supplementary Material

supp

## Figures and Tables

**Table 1:** Demographics and descriptive statistics of behavioral outcome.

Group	Age	Gender	Variant	FTLD-CDR Language	Baseline score	Immediately post-therapy - pre	2 weeks post therapy - pre	2 months post-therapy - pre
tDCS (n = 16)	64.3 (7.4)	8 M, 8F	8 lv, 4 nv, 4 sv	1.9 (0.8)	*Trained* 48.8 (29.3)	35.5 (25.9)	34.0 (25.6)	30.4 (24.9)
					*Untrained* 43.8 (26.9)	10.8 (11.3)	14.1 (16.3)	13.3(20.6)
Sham (n= 14)	68.8 (5.1)	7 M, 7F	6 lv, 5 nv, 3 sv	1.8 (0.8)	*Trained* 53.3 (22.7)	24.9 (18.1)	25.3(13.9)	14.1 (15.6)
					*Untrained* 55.9 (22.2)	7.6 (7.6)	12.1 (12.4)	1.4 (13.8)

*Note.* Demographic information and descriptive statistics of baseline letter accuracy (% of correctly spelled letters), and change in letter accuracy (mean and SD) for each post-therapy assessment time-point compared to the pre-therapy assessment. Sample size was reduced by 1 participant for Sham and 2 participants for tDCS at two weeks post-therapy and by 3 participants for tDCS at the 2-month post-therapy assessment, due to participant unavailability for testing.

**Table 2:** Predictors of the stimulation effect for trained and untrained words.

ROI	ΔR^2^	β	*p*
*Trained words: Immediately After therapy*			
Left Angular Gyrus	13.2%	−0.51	p < 0.01
Left Posterior Cingulate Cortex	14.3%	0.40	p < 0.05
*Untrained words: two months after therapy*			
Left Middle Frontal Gyrus	8.9%	−0.97	p < 0.001
Left Supramarginal Gyrus	4.6%	−0.39	p < 0.05
Right Posterior Cingulate Cortex	10.3%	0.38	p < 0.05

*Note:* For the remaining assessment times, adding volumetric data did not help improve the R^2^ of the model.

## Footnotes

4 Intracranial volume is similar to intracerebral volume but also includes the volumes of the myelencephalon and CSF.

5 We have conducted a separate study where we focus on behavioral predictors of the response to spelling therapy combined with tDCS (de Aguiar et al., *under review*).
