# Supplementary material for: Brain volumes as predictors of tDCS effects in primary progressive aphasia
Source: Brain Lang. Author manuscript; Available in PMC 2020 Dec 2. (PMC7709910; doi:10.1016/j.bandl.2019.104707)
Supplement: supp [file NIHMS1574729-supplement-supp.docx]

**Supplementary material 1 – supplementary information regarding data analyses**

In this study, our aim is to select brain regions that the volume (or the level of atrophy) can predict the tDCS effects on the language behavior outcome. Therefore, we considered the forward feature selection approach to first identify the brain regions that made a significant contribution in predicting the tDCS effect. After identifying brain regions, we fitted a multiple linear regression model to further elaborate the relationship. However, the number of brain regions is greater than the number of subjects. Thus, fitting a multiple linear regression with all brain regions is not reliable and a feature selection step is required. Hence, the analysis includes the following three steps.

**Step 1. Calculate the modified outcome.** In order to estimate the moderating effect of brain volume on the tDCS effect over sham, adapting the method proposed in Tian et al. (2014), we first calculate the modified outcome as

,

where is the primary outcome of follow-up of subject ; is the treatment assignment with for tDCS and for the sham; is the average of the primary outcome over subjects in the tDCS group and over sham group; is the proportion of subjects in the tDCS group.

**Step 2. Forward model selection.** In this step, we performed a forward model selection procedure based on the leave-one-out cross-validated coefficient of determination () using the modified outcomes from step 1. In the study, we imposed the pre-intervention score and global atrophy as a covariate kept in the model. The model selection was conducted on the brain volumetric data. This approach chose the brain region that gives the largest increment in the cross-validated , and was repeated until the increment is zero. The data were standardized first to avoid the scaling issue in the calculation.

**Step 3. Refit the regression model.** To further examine the moderating role of brain volumetric data on the tDCS effect, we refit the regression model with the modified outcome in step 1 as the dependent variable and pre-intervention score, global atrophy, and the brain regions selected in step 2 as the predictors. The model coefficients and the corresponding *p*-values were reported.

**Supplementary material 2 – descriptive statistics for brain volumes for each region of interest**

*Supplementary table.* Descriptive statistics for brain volumes (mm^3^) of the IFG (inferior frontal gyrus) and each region of interest identified as a predictor in the present study.
